# Environmental factors linked to depression vulnerability are associated with altered cerebellar resting-state synchronization

**DOI:** 10.1038/srep37384

**Published:** 2016-11-28

**Authors:** Aldo Córdova-Palomera, Cristian Tornador, Carles Falcón, Nuria Bargalló, Paolo Brambilla, Benedicto Crespo-Facorro, Gustavo Deco, Lourdes Fañanás

**Affiliations:** 1 Unidad de Antropología, Departamento de Biología Animal, Facultad de Biología and Instituto de Biomedicina (IBUB), Universitat de Barcelona; Av. Diagonal, 643, 08028, Barcelona, Spain; 2 Centro de Investigaciones Biomédicas en Red de Salud Mental (CIBERSAM); C/Doctor Esquerdo, 46, 28007, Madrid, Spain; 3 NORMENT, KG Jebsen Centre for Psychosis Research, Division of Mental Health and Addiction, Oslo University Hospital & Institute of Clinical Medicine, University of Oslo; Ullevål Hospital, Building 49, Kirkeveien 166, 0424, Oslo, Norway; 4 Center for Brain and Cognition, Computational Neuroscience Group, Department of Information and Communication Technologies, Universitat Pompeu Fabra; C/Roc Boronat, 138, 08018, Barcelona, Spain; 5 BarcelonaBeta Brain Research Center, Pasqual Maragall Foundation; C/Dr Aiguader, 88, 08003, Barcelona, Spain; 6 Centro de Investigación Biomédica en Red en Bioingeniería, Biomedicina y Nanomedicina (CIBER-BBN); C/Poeta Mariano Esquillor, s/n., 50018, Zaragoza, Spain; 7 Medical Image core facility, the Institut d’Investigacions Biomèdiques August Pi i Sunyer (IDIBAPS); C/Rosselló, 149-153, 08036, Barcelona, Spain; 8 Centro de Diagnóstico por Imagen, Hospital Clínico; C/Villarroel, 170, 08036 - Barcelona, Spain; 9 Department of Neurosciences and Mental Health, Psychiatric Clinic, Fondazione IRCCS Ca’Granda Ospedale Maggiore Policlinico, University of Milan; Via Francesco Sforza 35, 20122 Milano, Italy; 10 Department of Psychiatry and Behavioural Sciences, University of Texas Health Science Center at Houston; 7000 Fannin St #1200, Houston, TX 77030, United States; 11 University Hospital Marqués de Valdecilla, IDIVAL, Department of Medicine and Psychiatry, School of Medicine, University of Cantabria; Av. Valdecilla, s/n, 39008, Santander, Cantabria, Spain; 12 Institució Catalana de la Recerca i Estudis Avançats (ICREA), Universitat Pompeu Fabra; Passeig Lluís Companys, 23, 08010, Barcelona, Spain

## Abstract

Hosting nearly eighty percent of all human neurons, the cerebellum is functionally connected to large regions of the brain. Accumulating data suggest that some cerebellar resting-state alterations may constitute a key candidate mechanism for depressive psychopathology. While there is some evidence linking cerebellar function and depression, two topics remain largely unexplored. First, the genetic or environmental roots of this putative association have not been elicited. Secondly, while different mathematical representations of resting-state fMRI patterns can embed diverse information of relevance for health and disease, many of them have not been studied in detail regarding the cerebellum and depression. Here, high-resolution fMRI scans were examined to estimate functional connectivity patterns across twenty-six cerebellar regions in a sample of 48 identical twins (24 pairs) informative for depression liability. A network-based statistic approach was employed to analyze cerebellar functional networks built using three methods: the conventional approach of filtered BOLD fMRI time-series, and two analytic components of this oscillatory activity (amplitude envelope and instantaneous phase). The findings indicate that some environmental factors may lead to depression vulnerability through alterations of the neural oscillatory activity of the cerebellum during resting-state. These effects may be observed particularly when exploring the amplitude envelope of fMRI oscillations.

Although the cerebellum embodies only 10% of total brain mass, it hosts almost 70 billion neurons, nearly 80% of all neural cells in the human brain^1^. Through resting-state oscillatory activity, the cerebellum is functionally connected to large regions of the cerebral cortex, including not only the motor areas but also the prefrontal and parietal cortices^2^. These observations may partly explain the biological relevance of cerebellar resting-state activity disruptions observed across several psychiatric disorders^3^,^4^,^5^,^6^,^7^,^8^. Notably, recent research findings have consistently suggested cerebellar resting-state connectivity changes in depression, making it one of the best candidate mechanisms to elicit the neural alterations of the depressed brain^9^,^10^,^11^,^12^.

Although cerebellar resting state networks have not been extensively explored in the psychiatric literature on mood disorders, accruing evidence from recent studies suggests that they may be disrupted in the presence of depression liability. Putative explanatory mechanisms for such observations include the cerebellar functional connections to limbic regions such as the amygdala and the hippocampus, and the cerebellar role in cognitive-emotional processing^9^. Furthermore, novel data suggests that some cerebellar activity disruptions may be closely linked to the default mode network changes classically observed in depressive psychopathology^10^.

Both cerebellar resting-state connectivity and depression liability, when considered independently, are caused by the confluence of many genetic and environmental factors^13^,^14^,^15^. Although disengaging genes and environment has a prominent value in psychiatric research^16^, whether the association between cerebellar functionality and depression vulnerability can be explained by genetic or non-genetic factors remains largely unexplored. Genetically-informative studies need to be implemented to elicit this topic for two main reasons. First, it has been proposed that some alternative phenotypes (i.e., endophenotypes) would have a tougher connection with the genetic basis of psychopathology than phenomenologically-derived clinical diagnoses^17^,^18^. As some resting-state networks including cerebellar regions are likely to serve as endophenotypes in brain research^13^, it is feasible hypothesizing that some genetic factors determining cerebellar resting-state activity may also modify the risk for depression. Secondly, the link between the cerebellum and depression liability may perhaps be due to environmental factors. One could thus postulate that some environmental factors alter the cerebellar functionality and then lead to depression vulnerability. This would have significant implications as there are several well-identified environmental risk factors for depression whose underlying neurobiology has been only partly explained^19^,^20^. While the presence of resting-state fMRI alterations at cerebral regions –i.e., the default mode network– is broadly acknowledged in depressive disorders, the contribution of cerebellar function to those clinical phenotypes has recently been gaining attention^9^. This is a relevant point since data on healthy twins and families shows significant quantitative genetic components of whole-brain resting-state fMRI activity at cerebellar regions^13^,^14^, but their putative modulation of depression vulnerability remains only scarcely explored.

In this context, it is also worth mentioning that some specific genetic and environmental factors can induce neurobiological changes that lead to depressive psychopathology. For instance, polymorphic variation in genes such as the monoamine oxidase A (*MAOA*), the serotonin transporter (*5-HTT*) or the glucocorticoid receptor (*FKBP5*) can interact with early life stress and induce depression-related psychopathology^16^, mainly by sensitizing the hypotalamic-pituitary-adrenal axis. Additionally, several environmental factors are known to predispose to depressive disorders. Many of them take place during the infancy, such as growing in a low socio-economic status family, and experiencing childhood maltreatment and parental neglect/absence^20^. Furthermore, different adverse life events in adulthood, such as humiliation or the loss of a relative due to death or separation, can considerably increase the risk for depression^19^.

The former alterations in cerebellar resting-state connectivity observed in depressive psychopathology suggest a number of (potential) functional disruptions. Although using diverse neuroimaging techniques to assess resting-state patterns, several authors have agreed that there are many differences in cerebellar function between depression-prone and healthy individuals^9^. These different imaging analysis methods allow evaluating putative functional alterations from several viewpoints. While each of them might have its own potential relevance in clinical settings^21^, one of the most promising approaches to study resting-state neural activity *in vivo* is the examination of spatio-temporal synchrony patterns between regions using network theory^22^,^23^,^24^. In terms of large-scale neuronal networks, the brain disturbances reported in the literature of depression would indicate modifications of the information processing performance through the cerebellum.

Typically, the study of resting-state functional brain networks is based upon the extraction of low-frequency time series from a set of pre-defined anatomical regions. Robust first-order correlations in temporal activation patterns of two anatomically-segregated regions (nodes) are considered a functional connection (edge^23^,^25^). Highly synchronized (correlated) wave amplitudes of two brain regions during resting-state are thus interpreted as a strong edge linking those two regions. While this method has certainly led to remarkable clinical and neurobiological findings^21^, new reports highlight that the neural synchronization patterns observed through neuroimaging can occur at different levels, and that each of these levels may have different behavioral significance^26^,^27^. The potential relevance of the different neural coupling levels observed in resting-state fMRI brain scans has been underscored during recent years, since *i*) they allow increasing temporal resolution of hemodynamic (fMRI) signals^28^, *ii*) they may explain an important extent of the relationship between brain structure and function^29^ and *iii*) they could partly explain brain deficits in psychopathology^30^. A previous report on cerebral resting-state activity by our group^30^ described amygdalar deficits in twins with depression liability, highlighting the role of alternative neural coupling mechanisms not only to explain cerebral alterations, but also to elicit their genetic and environmental origins. In biological terms, these observations on synchronization phenomena observed in fMRI signals would parallel the compelling evidence showing that higher-order brain function may be closely related to neural communication established from coherent oscillatory activity of brain regions at specific frequencies^31^,^32^. Namely, distinct components of neuronal activity waves can encode and transmit information efficiently, although such components may not be directly deduced by examining raw fMRI time series.

Considering these elements, the present study was aimed at examining the putative association between cerebellar resting-state fMRI alterations and both the genetic and the environmental factors leading to depression liability. High-resolution fMRI brain scans were used to extract cerebellar resting-state time series from a group of 48 monozygotic (MZ) twins (24 pairs) informative for depression vulnerability. As co-twins of a MZ pair have almost identical DNA sequences, their phenotypic similarities and differences were investigated to gain insights on putative familial and environmental influences. Following conventions from classical quantitative genetic analysis, intrapair brain differences in genetically identical individuals (MZ twins) are considered a measure of environmental factors influencing that neural phenotype^13^,^30^. Since the cerebellar fMRI signal constitutes a well-defined resting-state network^33^, and considering that intra-cerebellar resting-state connectivity may be especially relevant in neuropsychiatry^34^, an anatomically-restricted 26-node network comprising different subdivisions of the cerebellum was examined. Functional connections (edges) between regions were defined using three complementary conceptions of neural synchrony^26^: *i*) amplitude correlation –the conventional method–, *ii*) amplitude envelope correlation and *iii*) phase synchrony.

## Results

As a preliminary step, intrapair correlations in global connectivity metrics describing the links between cerebellar subdivisions were analyzed. There were large intrapair differences within the MZ pairs across these measures (Table 1); the largest intrapair correlation coefficient for the different network metrics was 0.38, suggesting that the familial factors have only a moderate effect on those phenotypes, and that unique environmental influences may explain most of the observed variance. None of the correlation coefficients was statistically significant. Namely, co-twins from every MZ pair showed largely different cerebellar synchronization patterns. These environmental effects on cerebellar synchronization provided further support to the ensuing analyses separating the variance into genetic and non-genetic factors.

**Figure 1:**
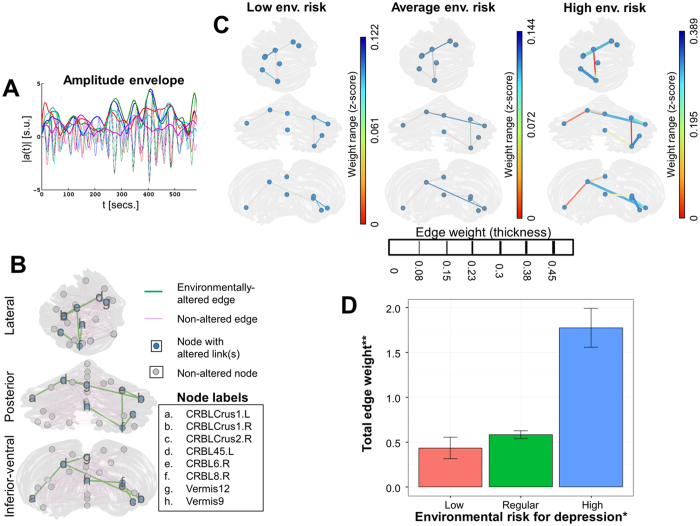
Environmental factors associated with depression vulnerability are linked to cerebellar synchronization disruptions. (**A**) The amplitude envelope obtained from the Hilbert-transformed resting-state signal allowed identifying a functional network in the cerebellum potentially altered due to environmental liability for depression. (**B**) A cerebellar synchronization sub-network comprising seven edges, built from oscillatory amplitude envelopes, was shown altered by the NBS approach. (**C**) There are marked network edge differences across individuals depending on environmental risk liability for depression. The leftmost plot corresponds to low environmental risk –averaged from five healthy co-twins from discordant pairs–, the plot in the middle depicts subjects with average environmental risk –average of five brains from randomly chosen concordant and healthy pairs–, and the rightmost plot shows participants with high environmental liability –five affected co-twins from discordant pairs–. (**D**) The barplot shows the mean and standard deviations of the total edge weights of the seven-edge network across the different environmental depression liabilities (red: low environmental risk; green: regular environmental risk; blue: high environmental risk). The values in the bars were retrieved by residualizing regression procedures from all 48 individuals, adjusting by age, gender and familial depression liability.

Next, network-based statistic (NBS) analyses were conducted to examine putative resting-state synchronization disruptions in the cerebellum of depression-prone subjects, dividing the depression liability into familial and unique environmental, and using three different time series analysis methods. When examining either the conventional approach (amplitude correlation) or the instantaneous phase, NBS analysis revealed no association between cerebellar resting-state activity and depression liability. In contrast, there were statistically significant results for the cerebellar resting-state network built from amplitude envelope correlations between the twenty-six regions of interest (Fig. 1). As noticed in Fig. 1, there was a seven-edge network that showed statistically significant differences in resting-state activation depending on the environmental liability for depression (NBS *p*-value for *β*_*W*_ of the sub-network: 0.002; *F* = 10.36). As described, the statistical significance of all the *p*-values retrieved from the NBS software tool is already controlling for the family-wise error rate (*Methods: Inter-subject analysis of the functional connectivity networks*). Although three different NBS analyses were implemented (conventional approach, amplitude envelope and instantaneous phase), the significance of the previous finding (*p* = 0.002) would persist even after an additional –and perhaps overly conservative– multiple testing adjustment stage (i.e., *p*_adjusted_ = 0.002 × 3 independent tests = 0.006).

Further exploratory analysis revealed that these network edges formed stronger connections between nodes in the individuals with high environmental load for depression (the affected co-twins from discordant pairs) than in the rest of the study population (Fig. 1). This suggests that an increased environmental liability for psychopathology would be related to hyper-synchronized activity across a set of cerebellar ROIs, including portions of the left and right crura, parts of the vermis and other subdivisions of both cerebellar hemispheres.

Since the NBS approach does not provide a direct adjust for heteroscedasticity, and in order to obtain further insights on the results for this set of edges, a confirmatory procedure was conducted. Namely, a linear regression model (*Methods: Inter-subject analysis of the functional connectivity networks*) was implemented to analyze the seven cerebellar edges shown altered by NBS. This analysis suggested that the previous finding is statistically robust even after adjusting for the correlated nature of twin data (familial factors: *β*_*B*_ = 0.11, standard error = 0.17, *t* = 0.62, *p* = 0.541; unique environment: *β*_*W*_ = 1.34, standard error = 0.29, *t* = 4.71, *p* < 0.0001; adjusted *R*^2^ for the whole model: 0.45). The results of this step are depicted in the barplot of Fig. 1, which shows that, after controlling for gender and age, the affected co-twins from discordant pairs have more than twice the total network edge weight than the rest of the participants. Importantly, this result remained statistically significant after removing the six individuals with predominantly anxious psychopathology (familial factors: *β*_*B*_ = 0.19, standard error = 0.2, *t* = 0.96, *p* = 0.342; unique environment: *β*_*W*_ = 1.28, standard error = 0.31, *t* = 4.16, *p* = 2*10^−4^; adjusted *R*^2^ for the whole model: 0.42).

## Discussion

A genetically-informative design was implemented here to evaluate the putative relationship between depression liability and different cerebellar resting-state synchronization patterns, as measured by fMRI. Three different coupling types (amplitude, amplitude envelope and instantaneous phase correlation) among the distinct anatomical subdivisions of the cerebellum were analyzed in relation to both familial and unique environmental factors underlying depression liability. Overall, there were large differences in cerebellar synchronization –at all three levels– within MZ twin pairs, likely related to environmental factors. When considering the amplitude envelope of the resting-state fMRI activity patterns, the temporal correlations between paired cerebellar ROIs showed an association with the environmental factors leading to depression vulnerability. In contrast, depression risk –either familial or environmental– was not related to the cerebellar coupling patterns of either the amplitude or the instantaneous phase. To the best of our knowledge, this is the first genetically-informative study of cerebellar resting-state functional networks in depression-prone individuals examining not only the network formed by BOLD wave amplitudes, but also those derived from their analytic components.

The results suggest that some analytic properties of the resting-state fMRI BOLD signal may be associated with differential exposure to environmental factors leading to depression vulnerability. More specifically, when analyzing the amplitude envelope of the resting-state fMRI patterns, individuals with high environmental risk load for depression showed a set of overly synchronized cerebellar regions. This hyper-synchronization pattern is analogous to other neural coupling impairments observed across different neuropsychiatric pathologies. For example, the complexity of the neural oscillatory activity is decreased in schizophrenia, as indexed by high synchrony between different cerebral regions^35^,^36^,^37^. Perhaps the boundary expression of neural hyper-synchronization leading to a functional impairment is observed in the epileptic brain^38^,^39^. Physically, the redundancy in information through different sources (i.e., ROIs having very similar oscillatory patterns) is linked to a reduction in communicational complexity^40^.

The fact that the observed effects are mainly associated with environmental factors should be emphasized, particularly since there is evidence of important environmentally-induced physiological changes in the cerebellum^41^,^42^,^43^,^44^. These non-genetic factors may explain part of the relatively high influence of the environment on depression liability (i.e., an heritability estimate around 40%)^15^. Although the evidences on the environment and the cerebellum come mainly from animal research developed in laboratory settings, extensive epidemiological literature has demonstrated a significant role for specific environmental factors leading to depression in humans. Interestingly, non-genetic factors typically associated with adult depression, such as early stressful experiences, have recently been suggested in a literature review as potential modifiers of the cerebellar functionality^45^.

Some potential limitations deserve mention. First, the present report may not be directly comparable to a large extent of the brain network literature, since most studies implement parcellation schemes excluding the cerebellum^46^,^47^, and high-resolution fMRI scans are needed in order to map all its subdivisions correctly. This limitation is not specific to the current report; the choice of parcellation schemes is a key subject with enormous consequences for brain connectomics^48^. Nevertheless, the inclusion of 26 cerebellar ROIs as the very focus of this study may have improved the specificity of the findings. Future works may combine finer-grained parcellations with higher-resolution neuroimaging scans. Furthermore, the sample size was relatively small. However, having found strong associations suggests the presence of relatively robust effects. Finally, limitations regarding the clinical features of the ongoing sample should be addressed in later studies. For instance, six subjects with mostly anxious psychopathology were included. Even though the reported associations persisted after repeating the analyses without those subjects, complementary research designs may improve the specificity of the findings by considering narrower phenotypic categories. Similarly, samples with different severities of transversally-measured symptoms could enhance the generalizability of the findings. Due to these points, confirmatory evidence is needed to strengthen the conclusions.

Overall, these results indicate that the non-genetic factors leading to depression vulnerability are associated with disrupted cerebellar synchronization. They also point out that different resting-state cerebral phenotypes –obtained using different time-series analysis techniques– may or not be linked to particular behaviors. When examining the neurobiological correlates linking an environmental exposure with depression, it might be appropriate using the amplitude envelop of low-frequency resting-state fMRI BOLD oscillations to build biological networks from the cerebellum.

## Methods

### Sample description

The participants of this study were selected from a larger group of 115 Spanish Caucasian adult twin pairs (230 individuals) from the general population, who gave their consent to be contacted for research purposes. All the subjects were contacted by telephone and invited to participate in a general study of adult cognition and psychopathology. Trained psychologists administered a battery of neurocognitive and psychological tests to the twins. Also, they were interviewed for medical records. Exclusion criteria applied were a medical history of neurological disturbance, presence of sensory or motor alterations, current substance misuse or dependence and age under 18 and over 65 years. Written informed consent was obtained from all participants after a detailed description of the study aims and design. The institutional ethics committee (Comissió de Bioètica de la Universitat de Barcelona (CBUB); Institutional Review Board registry IRB00003099; Assurance number: FWA00004225; http://www.ub.edu/recerca/comissiobioetica.htm) approved the written informed consent and the overall study. The methods were carried out following the approved guidelines, which were in accordance with the Declaration of Helsinki.

Zygosity of all pairs was evaluated by genotyping 16 highly polymorphic microsatellite loci from DNA samples (SSRs; PowerPlex^®^ 16 System Promega Corporation). Identity on all the markers can be used to assign monozygosity with greater than 99% accuracy^49^. In the whole sample (115 duos), 86 twin pairs were MZ.

From the former collection of participants, using the previously obtained data, a subset of 54 individuals (27 MZ twin pairs) was selected, as they were informative for psychopathological traits and agreed to participate in the MRI part of the present study. These 54 participants met the following criteria: *i*) age at scan between 20 and 56 years, *ii*) both twins right-handed, and *iii*) none of the twins manifested liability for DSM-IV-R psychiatric diagnoses other than depression and/or anxiety. No participant had a history of major medical illnesses. Due to image artifacts or lack of data for six participants, the final sample (i.e., the subset included in all statistical analyses) consisted of 48 individuals (20 males, mean age: 33.6 years).

### Psychometric measures

A clinical psychologist in a face-to-face interview evaluated the liability for psychopathology in this general population sample. Briefly, the Structural Clinical Interview for DSM-IV Axis I Disorders (SCID-I)^50^ was applied in a face-to-face interview to screen for the presence of any lifetime depression or related anxiety spectrum disorder. In this sample, six individuals with a history of (mainly) anxious psychopathology were included in the psychopathology-affected group. This apparently wide category of outcomes was used in recognition of the evidence on comorbidity, shared etiopathology and diagnostic criteria overlap between depressive and anxious disorders^51^,^52^,^53^,^54^, as well as considering findings of some similar resting-state alterations across both diagnoses^55^,^56^. The distribution of lifetime and current diagnoses of psychopathology in the participants was as follows: lifetime depression and lifetime anxiety (*n* = 5), lifetime depression (*n* = 4), lifetime anxiety (*n* = 3), actual depression and lifetime depression (*n* = 2), actual anxiety and lifetime anxiety (*n* = 2), actual depression (*n* = 1), actual depression and lifetime anxiety (*n* = 1), lifetime depression and actual anxiety (*n* = 1), and actual anxiety (*n* = 1). Of note, repeating the statistical analyses removing predominantly anxious individuals (lifetime anxiety, actual anxiety and lifetime anxiety, and actual anxiety, totaling *n* = 6) did not alter the significance of the results.

The participants were also asked to report if they had received psychological or pharmacological treatment or had consulted a mental health professional since they first participated in the study. Only one individual had life-time exposure to psychopharmacological treatment for depression. However, excluding this individual from the group analyses did not change the significance of the results.

In the whole sample, there were ten healthy, six concordant and eight discordant pairs for lifetime DSM-IV diagnoses. Furthermore, current depression status and other psychiatric symptoms were evaluated using the Brief Symptom Inventory (BSI^57^,^58^). The BSI is a self-administered 46-item screening instrument designed to identify the experience of psychopathological symptoms during the last 30 days. It includes six subscales (depression, phobic anxiety, paranoid ideation, obsession-compulsion, somatization and hostility) and is designed for use in both clinical and non-clinical samples. Items are rated on a five-point scale of distress, according to self-perception of symptom severity. Twins with no lifetime history of DSM-IV diagnosis had fewer self-reported symptoms –lower BSI scores– in both the depression subscale and the whole questionnaire (Table 2). Moreover, neurocognitive information for this sample was collected by means of the Wechsler Adult Intelligence Scale^59^,^60^. The intelligence quotient (IQ) of each participant was estimated from five subtests of this battery (block design, digit span, matrix reasoning, information and vocabulary). The distribution of IQ scores was similar to those reported in demographically analogous samples^61^ (Table 2). As no intra-group differences in IQ scores were found, neurocognitive effects on resting-state brain signals^62^,^63^ are not likely to confound the statistical analyses in this study.

### MRI acquisition and pre-processing

The brain scans were acquired at the MRI Unit of the Image Platform (IDIBAPS, Hospital Clínic de Barcelona), using a TIM TRIO 3 T scanner with an 8-channel head coil (Siemens, Erlangen, Germany). The resting-state fMRI images comprised 210 echo-planar (EPI) blood-oxygen-level dependent (BOLD) sensitive volumes (TR = 2790 ms, TE = 30 ms, 45 axial slices parallel to anterior-posterior commissure plane acquired in interleaved order, 3.0 mm slice thickness and no gap, FOV = 2075 × 1344 mm^2^, voxel size = 2,67 × 2,67 × 3 mm^3^). Moreover, high-resolution 3D structural datasets were acquired for anatomical reference, using a T1-weighted magnetization prepared rapid gradient echo, with the next parameters: 3D T1-weighted MPRAGE sequence, TR = 2300 ms, TE = 3.03 ms, TI = 900 ms, Flip Angle = 9°, 192 slices in the sagittal plane, matrix size = 256 × 256, 1 mm^3^ isometric voxel.

**Figure 2:**
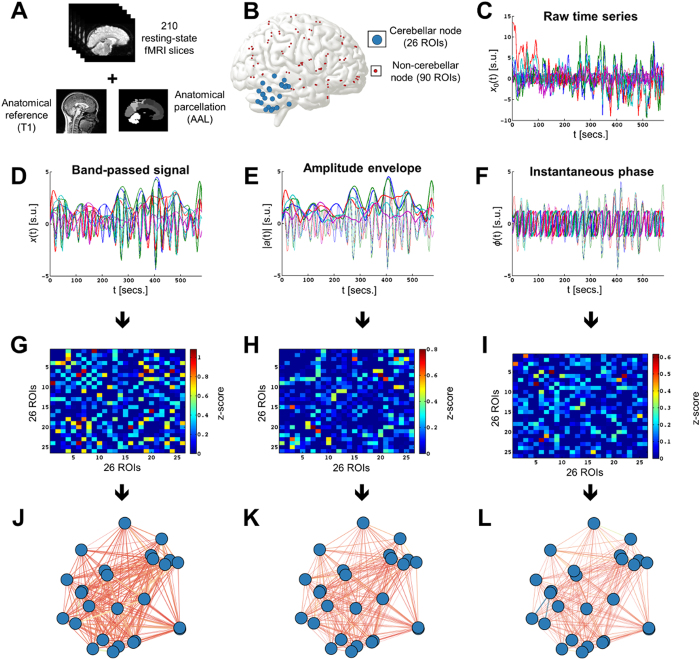
Schematic representation of the construction of three functional networks for one cerebellum. (**A**) The 210 resting-state fMRI volumes (slices) are co-registered to the anatomical T1 3D reference volume, and each voxel is mapped to one of the 116 ROIs in the AAL atlas (including the cerebellum). (**B**) The anatomical atlas allows segmenting the brain into 90 cerebral and 26 cerebellar ROIs, and after artefact removal, a time series of the mean (BOLD) activation probability for each of the 116 ROIs is obtained. (**C**) For each ROI, a raw time series is retrieved using the 210 fMRI slices acquired through 9:56 minutes of scan. (**D**) A band-pass filter is applied to obtain the resting-state fMRI narrowband signal (0.04–0.07 Hz). (**E**) The amplitude envelope of each band-passed wave is estimated for later analysis. (**F**) Similarly, the Hilbert transform also allows calculating the instantaneous phase of the waves. (**G**–**I**) Three partial correlation matrices are obtained from the previous time-series (band-passed and Hilbert-transformed amplitude envelope and phase); they are z-transformed to normalize correlation values across individuals. Warm (cold) colours in these matrices represent large (small) correlation values between ROIs. The left tail of these correlation matrices (i.e., edges with negative z-scores) are set to 0 following a soft-thresholding procedure. (**J**–**L**) Graph-theoretical measures of edge weight are obtained for each pair of cerebellar regions, to be analyzed using NBS.

Resting-sate time series were obtained using standard image processing protocols implemented in the Statistical Parametric Mapping software, version 8 (SPM8^64^), running under MATLAB (The Mathworks, Natick, MA). After correction of slice-timing differences and head-motion, the fMRI images were co-registered to the 3D (T1) anatomical reference and to the mean functional image. Then, the images were spatially normalized to the standard stereotaxic space MNI^65^. Artifacts related to blood pulsation, head movement and instrumental spikes were removed from the BOLD time series in MNI space, using independent component analysis as implemented in GIFT^66^,^67^. The full cerebellum was properly scanned in the acquisition field of view. To improve test-retest reliability, no global signal regression was conducted^68^; no spatial smoothing was applied, in order to avoid adding spurious correlations between adjacent voxels^34^. Mean BOLD time series were extracted from the 116 regions of interest (ROIs) in the standard Automatic Anatomical Labeling (AAL) atlas, which comprises 90 cerebral and 26 cerebellar regions^47^. A description of all cerebellar ROIs included in the AAL atlas can be found in *Supplementary Material*. The atlas had previously been masked with the binarized subjective tissue probability maps to detach the mean value of the regions from the gray matter via a conventional protocol^69^,^70^. The following mask was used: [Atlas * (GM > WM) * (GM > CSF) * (GM > 0.1)], where GM stands for gray matter, WM is the white matter and CSF stands for cerebrospinal fluid. Next, the BOLD time series for each region were band-pass filtered within the resting-state fMRI narrowband going from 0.04 to 0.07 Hz^28^,^71^ (Fig. 2).

### Extraction of functional connectivity networks for each individual

Three different approaches were used here to estimate functional connectivity from the band-passed time series described above. First, a conventional approach to examine correlations between fMRI BOLD time series was used^24^, with twenty-six *x*(*t*) series per individual (one for each cerebellar AAL ROI). A partial correlation matrix was obtained for the 26 ROIs from the 210 slices scanned over time. Each partial correlation coefficient from this matrix represents the magnitude of the association between every pair of ROIs, controlling for the effect of the other variables (i.e., the remaining ROIs). This step produced a 26 × 26 matrix representing the functional connectivity between each pair of brain ROIs, which was then normalized using Fischer’s z transform^72^,^73^. Subsequently, following a previous technical report^74^, negative edges were removed using soft thresholding, since their particular network topology can drastically alter the properties of brain fMRI connectivity networks (Fig. 2).

Other two functional connectivity networks were retrieved for each participant. In order to get them, the analytic components of the resting-state BOLD signals from the 26 ROIs were computed following previously published protocols^26^,^28^. Briefly, the analytic representation of each real valued band-passed (0.04–0.07 Hz) BOLD time series was computed by further processing their band-passed time series, using the Hilbert transform. Explicitly, let *x*(*t*) be the band-passed BOLD time series of a particular ROI. Its analytical representation is the complex function


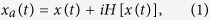


where *i* stands for 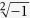, and *H*[·] is the Hilbert transform. The new signal *x*_*a*_(*t*) has the same Fourier transform as *x*(*t*), although it is defined only for positive frequencies. Similarly, let *x*(*t*) be expressed as an amplitude-modulated signal *a*(*t*) with carrier frequency *ϕ*(*t*), so that *x*(*t*) = *a*(*t*)cos[*ϕ*(*t*)]. Then, its Hilbert transform gives


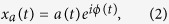


where |*a*(*t*)| represents the instantaneous envelope and *ϕ*(*t*) stands for the instantaneous phase. In the present study, both |*a*(*t*)| and *ϕ*(*t*) are later used to estimate two new 26 × 26 partial correlation matrices as described above, which are later z-transformed and soft-thresholded (Fig. 2).

### Inter-subject analysis of the functional connectivity networks

A network-based statistic (NBS) approach^75^ was implemented to examine potentially altered connections (edges) between every pair of ROIs (nodes). Briefly, NBS performs a statistical examination of potentially altered network edges that may differ across groups or conditions. It controls the family-wise error rate when statistical tests are conducted at single edges comprising a whole graph, on the basis of conventional cluster-based thresholding of statistical parametric maps^75^. Since edge weights in the connectivity matrices studied here represent oscillatory coupling events between pairs of ROIs, the edge-based strategy of NBS allowed assessing potential coupling alterations across combinations of cerebellar regions. These oscillatory patterns were examined through independent analyses, implemented for each of the three partial correlation matrices: amplitude, amplitude envelope and instantaneous phase (corresponding to *x*(*t*), |*a*(*t*)| and *ϕ*(*t*)).

The design matrices used for NBS were based upon previous literature on regression models of clustered data^76^, which can be applied to separate familial and environmental components of phenotype associations in twin studies^77^. More explicitly, the regression model


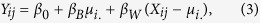


was implemented. Subindex *i*∈{1, …, *n*} stands for pair number (here, *n* = 24 MZ pairs) and *j*∈{1, 2} refers to co-twin number (randomly assigned). The diagnostic status was coded as the numeric value 0 (healthy) or 1 (depressed) for each individual. An individual’s diagnostic is thus represented as *X*_*ij*_∈{0, 1} in the equation, and a pair’s familial liability for depression is expressed as *μ*_*i*._ = (*X*_*i*1_ + *X*_*i*2_)/2. The binary codification of the diagnostic status thus allows the familial liability (genes plus shared environment) to take only three values *μ*_*i*._∈{0, 0.5, 1}, corresponding to no familial liability (healthy pairs, *μ*_*i*._ = 0), moderate familial load (discordant pairs, *μ*_*i*._ = 0.5) or high familial predisposition (concordant pairs, *μ*_*i*._ = 1). This value is then used to estimate a regression coefficient for the familial factors (*β*_*B*_). Furthermore, the value *X*_*ij*_ − *μ*_*i*._ is computed to represent the unique environmental influences from non-shared events within a twin pair (*β*_*W*_). This arithmetic difference may only take the values 0.5, −0.5 and 0, as follows: *X*_*ij*_ − *μ*_*i*._ = 1−0.5 = 0.5 (high environmental risk: affected co-twins from discordant pairs), *X*_*ij*_ − *μ*_*i*._ = 0−0.5 = −0.5 (low environmental risk: healthy co-twins from discordant pairs), *X*_*ij*_ − *μ*_*i*._ = 1−1 = 0 (average environmental risk: concordant pairs), and *X*_*ij*_ − *μ*_*i*._ = 0−0 = 0 (average environmental risk: healthy pairs). Namely, both concordant and healthy pairs are assumed to have no environment-specific differences in depression liability, whereas affected (healthy) discordant co-twins are considered to have high (low) environmentally-induced risk. This variable is intended to reflect the fact that, in discordant pairs, the affected co-twin was exposed to the environmental risk factor (*high* environmental risk), whereas his/her healthy co-twin was not (*low* environmental risk). Lastly, *Y*_*ij*_ represents the edge weight of each connection between the 26 different nodes in the cerebellum. Thus, the equation is solved for all edges separately, although the method implemented by NBS already exploits the fact that the connections with an effect of interest may usually be interconnected^75^. Moreover, to control for potential confounding demographics (Table 2), all analyses were adjusted for gender and age.

Complementarily, a confirmatory analysis was conducted using R’s software packages *rms* and *mztwinreg*^78^,^79^,^80^. Namely, the cerebellar subgraph comprising the altered edges from the above procedure was retrieved, and total edge weight –a global measure of oscillatory synchronization of the potentially-altered connections– was computed. This value (*Y*_*ij*_) was used as outcome in the equation mentioned above. It was then solved via ordinary least squares, and the Huber-White method was implemented to adjust the variance-covariance matrix of these regression fits, in order to account for the non-independence of twin data (heteroskedasticity).

## Additional Information

**How to cite this article**: Córdova-Palomera, A. *et al*. Environmental factors linked to depression vulnerability are associated with altered cerebellar resting-state synchronization. *Sci. Rep.* **6**, 37384; doi: 10.1038/srep37384 (2016).

**Publisher’s note:** Springer Nature remains neutral with regard to jurisdictional claims in published maps and institutional affiliations.

## Supplementary Material

Supplementary Material

## Figures and Tables

**Table 1:** Edge-based parameters describing the twenty-six-node cerebellar networks.

	Individual Level					
	Conventional (amplitude correlation)^a^		Amplitude envelope correlation^b^		Instantaneous phase correlation^c^	
	Mean (S.D.)	Range	Mean (S.D.)	Range	Mean (S.D.)	Range
Total edge weight	53.69 (4.51)	45.75–64.55	31.54 (2.18)	27.71–36.47	21.67 (2.13)	16.52–27.41
Average edge weight (connected)^d^	0.31 (0.03)	0.25–0.39	0.18 (0.01)	0.16–0.21	0.12 (0.01)	0.1–0.16
Average edge weight (all cells)^e^	0.16 (0.01)	0.13–0.19	0.09 (0.01)	0.08–0.11	0.06 (0.01)	0.05–0.08
Maximum edge weight	1.12 (0.23)	0.79–2.21	0.81 (0.51)	0.54–3.94	0.57 (0.22)	0.36–1.87
	**Intrapair Differences**					
	**Spearman’s Rho**	***p*-value**	**Spearman’s Rho**	***p*-value**	**Spearman’s Rho**	***p*-value**
Total edge weight	−0.14	0.5	0.09	0.66	0.25	0.25
Average edge weight (connected)	−0.17	0.42	0.28	0.19	0.25	0.25
Average edge weight (all cells)	−0.38	0.07	0.15	0.48	−0.07	0.73
Maximum edge weight	−0.14	0.5	0.09	0.66	0.25	0.25

Three different approaches were used to build fMRI connectivity networks. First, the amplitude correlation method for band-passed low-frequency oscillations^24^ (a) afterward, the Hilbert-transformed signal allowed extracting the amplitude envelope (b) and the instantaneous phase (c) correlation methods^28^,^26^. The results indicate edge weights considering both the connected network component, removing all zero entries of the matrix (d), and all edges accounting for the zeroed matrix entries (e). S.D., standard deviation.

**Table 2:** Demographic, psychopathological and neurocognitive data for DSM-IV diagnostic concordant, discordant and healthy MZ twin pairs.

	Concordant (12 subjects, 10 female)		Discordant (16 subjects, 10 female)		Healthy (20 subjects, 8 female)		Group comparison
	Mean (SD)	Range	Mean (SD)	Range	Mean (SD)	Range	X-squared^a^; *p*
Age	42.5 (13)	22–54	37 (10.9)	20–50	30.3 (7.3)	19–39	5.9; 0.052
IQ	105.1 (12.5)	87–127	108.1 (11.8)	87–131	110.5 (5.5)	103–118	1.9; 0.393
Current psycho- pathology (total BSI)	27.9 (16.5)	6–57	20.9 (13.3)	4–45	10.6 (9.3)	1–33	8.7; 0.013*
Current depressive symptoms (BSI subscale)	6.9 (6.5)	1–20	3.5 (2.7)	0–9	1.7 (1.8)	0–6	6.4; 0.04*

Notes: SD, standard deviation; IQ, intellectual quotient; BSI, Brief Symptom Inventory.

^a^Kruskal-Wallis X-squared, as these variables were continuous.

^*^Statistically significant *p*-value.
