# Supplementary material for: Environmental factors linked to depression vulnerability are associated with altered cerebellar resting-state synchronization
Source: Sci Rep. 2016 Nov 28;6:37384. doi: 10.1038/srep37384 (PMC5124945; doi:10.1038/srep37384)

Supplementary material

The next material refers to the manuscript:

Environmental factors linked to depression vulnerability are associated with altered cerebellar resting-state synchronization

Authored by:

Aldo Córdova-Palomera[a], Cristian Tornador[a], Carles Falcón, Nuria Bargalló, Paolo Brambilla, Benedicto Crespo-Facorro, Gustavo Deco[b], Lourdes Fañanás[b]

[a] ACP and CT contributed equally to this work.
[b] GD and LF contributed equally to this work.

Page

Supplementary Table 1 2

Supplementary Figure 1 3

SUPPLEMENTARY TABLE 1. Description of the 26 cerebellar ROIs from the AAL atlas.

Notes: *, MNI coordinates; **, two standard sets of lables. Descriptions of the regions of interest (ROIs) were extracted from http://neuro.imm.dtu.dk/wiki/Automated_Anatomical_Labeling. The MNI coordinates represent the centroids of each ROI. See also Supplementary Figure 1.

ID	x*	y*	z*	Label 1**	Label 2**	Description
1	-36.067	-66.72	-28.934	CRUS1.L	Cerebelum_Crus1_L	Left crus I of cerebellar hemisphere
2	37.456	-67.137	-29.547	CRUS1.R	Cerebelum_Crus1_R	Right crus I of cerebellar hemisphere
3	-28.636	-73.26	-38.204	CRUS2.L	Cerebelum_Crus2_L	Left crus II of cerebellar hemisphere
4	32.057	-69.016	-39.949	CRUS2.R	Cerebelum_Crus2_R	Right crus II of cerebellar hemisphere
5	-8.8004	-37.223	-18.581	CB3.L	Cerebelum_3_L	Left lobule III of cerebellar hemisphere
6	12.319	-34.466	-19.391	CB3.R	Cerebelum_3_R	Right lobule III of cerebellar hemisphere
7	-15.004	-43.486	-16.933	CB4_5.L	Cerebelum_4_5_L	Left lobule IV, V of cerebellar hemisphere
8	17.199	-42.861	-18.151	CB4_5.R	Cerebelum_4_5_R	Right lobule IV, V of cerebellar hemisphere
9	-23.238	-59.101	-22.131	CB6.L	Cerebelum_6_L	Left lobule VI of cerebellar hemisphere
10	24.691	-58.316	-23.645	CB6.R	Cerebelum_6_R	Right lobule VI of cerebellar hemisphere
11	-32.358	-59.82	-45.449	CB7b.L	Cerebelum_7b_L	Left lobule VIIB of cerebellar hemisphere
12	33.139	-63.178	-48.457	CB7b.R	Cerebelum_7b_R	Right lobule VIIB of cerebellar hemisphere
13	-25.751	-54.519	-47.685	CB8.L	Cerebelum_8_L	Left lobule VIII of cerebellar hemisphere
14	25.064	-56.34	-49.468	CB8.R	Cerebelum_8_R	Right lobule VIII of cerebellar hemisphere
15	-10.947	-48.95	-45.903	CB9.L	Cerebelum_9_L	Left lobule IX of cerebellar hemisphere
16	9.4602	-49.5	-46.327	CB9.R	Cerebelum_9_R	Right lobule IX of cerebellar hemisphere
17	-22.614	-33.8	-41.765	CB10.L	Cerebelum_10_L	Left lobule X of cerebellar hemisphere
18	25.995	-33.838	-41.347	CB10.R	Cerebelum_10_R	Left lobule X of cerebellar hemisphere
19	0.75743	-38.792	-20.05	VERMIS1_2	Vermis_1_2	Lobule I, II of vermis
20	1.3804	-39.931	-11.398	VERMIS3	Vermis_3	Lobule III of vermis
21	1.2207	-52.362	-6.1138	VERMIS4_5	Vermis_4_5	Lobule IV, V of vermis
22	1.1414	-67.059	-15.123	VERMIS6	Vermis_6	Lobule VI of vermis
23	1.1458	-71.93	-25.141	VERMIS7	Vermis_7	Lobule VII of vermis
24	1.1521	-64.429	-34.08	VERMIS8	Vermis_8	Lobule VIII of vermis
25	0.86467	-54.875	-34.896	VERMIS9	Vermis_9	Lobule IX of vermis
26	0.35584	-45.8	-31.683	VERMIS10	Vermis_10	Lobule X of vermis

SUPPLEMENTARY FIGURE 1. Network nodes from the cerebellar ROIs in the AAL atlas.

Notes: The labels shown correspond to the column "Label 2" in Supplementary Table 1.

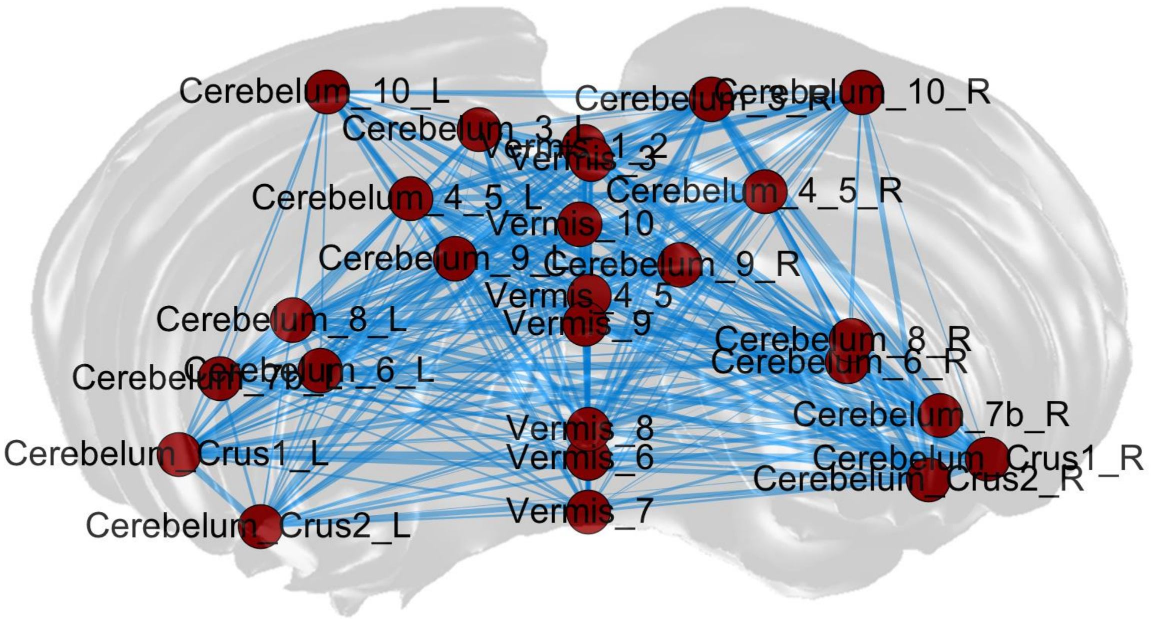

Supplement: Supplementary Material [file srep37384-s1.pdf]
